# Transcriptionally promiscuous “blurry” promoters in Tc1/*mariner* transposons allow transcription in distantly related genomes

**DOI:** 10.1186/s13100-019-0155-6

**Published:** 2019-04-03

**Authors:** Antonio Palazzo, Patrizio Lorusso, Csaba Miskey, Oliver Walisko, Andrea Gerbino, Carlo Marya Thomas Marobbio, Zoltán Ivics, René Massimiliano Marsano

**Affiliations:** 1 Department of Biology, University of Bari “Aldo Moro”, via Orabona 4, 70125 Bari, Italy; 2 Transposition and Genome Engineering, Division of Medical Biotechnology, Paul Ehrlich Institute, Langen, Germany; 3 Department of Biosciences, Biotechnologies and Biopharmaceutics, University of Bari, 70125 Bari, Italy; 4 Present address: Laboratory of Translational Nanotechnology, “Istituto Tumori Giovanni Paolo II” I.R.C.C.S, Viale Orazio Flacco 65, 70125 Bari, Italy

**Keywords:** Horizontal gene transfer, Promoter, *Tc1/mariner* transposons, Luciferase assay, transcriptional regulation, transposition, D, Melanogaster, S, Cerevisiae, H, Sapiens, E, Coli

## Abstract

**Background:**

We have recently described a peculiar feature of the promoters in two *Drosophila Tc1*-like elements, *Bari1* and *Bari3*. The AT-richness and the presence of weak core-promoter motifs make these promoters, that we have defined “blurry”, able to activate transcription of a reporter gene in cellular systems as diverse as fly, human, yeast and bacteria. In order to clarify whether the blurry promoter is a specific feature of the *Bari* transposon family, we have extended this study to promoters isolated from three additional DNA transposon and from two additional LTR retrotransposons.

**Results:**

Here we show that the blurry promoter is also a feature of two vertebrate transposable elements, *Sleeping Beauty* and *Hsmar1*, belonging to the *Tc1/mariner* superfamily. In contrast, this feature is not shared by the promoter of the *hobo* transposon, which belongs to the hAT superfamily, nor by LTR retrotransposon-derived promoters, which, in general, do not activate transcription when introduced into non-related genomes.

**Conclusions:**

Our results suggest that the blurry promoter could be a shared feature of the members of the *Tc1/mariner* superfamily with possible evolutionary and biotechnological implications.

**Electronic supplementary material:**

The online version of this article (10.1186/s13100-019-0155-6) contains supplementary material, which is available to authorized users.

## Background

Transposable elements (TEs) are widespread genetic elements that have played a fundamental role in genome evolution [1], contributing to generating diversity, both at small and large scale [2], and to evolving new functions through molecular domestication [3] or exaptation [4]. Their ubiquitous presence in the genomes of extant species suggests an ancient history dating back to early living organisms, as well as an extraordinary ability to overcome the canonical genetic barriers between species, the latter being an intrinsic feature of the horizontal gene transfer process (HGT).

TEs, like other genes, are usually transmitted from parents to offspring, and propagate in the population. In parallel to one or several bursts of genomic expansion TEs are subjected to mutational load, denoting their a neutral mode of evolution. The overall absence of selection acting on TEs establishes a kind of “genomic homeostasis”, i.e. a balance in terms of numbers of functional and non-autonomous TE copies. When mutant copies overcome functional ones, the TE is destined to extinction in the genome in which it resides, an effect that could be also translated at the species level [5, 6]. Horizontal Transposon Transfer (HTT) is an important mechanism that mobile genetic elements undertake to escape extinction.

*Tc1* and *mariner* are two related DNA transposon families, part of the *Tc1/mariner/pogo/IS630* (typically referred to as *Tc1/mariner* in short) superfamily. They were first identified in nematode [7] and insect [8] genomes respectively, and are both characterized by an open reading frame encoding a transposase flanked by two terminal inverted repeats (TIRs) and TA dinucleotides representing duplicated target sites [9]. Previous studies have identified *Tc1*-like elements in a variety of animals and fungi [10], as well as in the parasitic amoebozoa *Entamoeba invadens* [11] and in plants [12]. The ubiquitous presence of *Tc1/mariner*-like elements in the genomes of virtually all extant eukaryotic species and the phylogenetic inconsistencies found in many cases studied [13, 14] support the hypotheses that they are ancient components of the eukaryotic genomes and could have also spread by means of HTT [15].

The establishment of a TE in a new genome after a HTT event depends on the expression of the TE-encoded genes required for element mobilization and propagation. Considering that expression of such genes relies to a large extent on the activity of their own promoters, it can be predicted that the greater the evolutionary distance between the donor and recipient species involved in the HTT event, the lesser the chance that the promoter could be recognized by transcription factors of the new host. Translated in terms of transposition efficiency, insufficient expression of transposition-related proteins would mean that the TE will be “dead on arrival” in the new host unless other surviving strategies are adopted.

We have recently reported that the promoter elements isolated from two related *Drosophila Tc1*-like transposons, *Bari1* and *Bari3*, are functional in evolutionarily distant genomic backgrounds [16]. These transposons carry an AT-rich “blurry promoter” with divergent or no sharply predictable core-promoter motifs, which is able to drive transcription when transplanted into an unrelated genetic and genomic context. On the basis of our previously reported results, we hypothesized that this feature could be important for the success of HTT events of such genetic elements. The TF/TFBS (Transcription Factor/Transcription Factor Binding Site) recognition is indeed the result of complex co-evolution processes [17] that does not allow promoters to work properly in divergent genetic backgrounds. Our previous results [16], based on the comparison of the promoters of the *Bari1* and *Bari3* transposons with that of an LTR (Long Terminal Repeat) retrotransposon (the *copia* element), suggested that the blurry promoter is a peculiarity of the *Tc1* family.

Here, in order to further investigate the existence of the blurry promoter in *Tc1*-like elements from other organisms and to assess whether *mariner*-like elements also have similar feature, we extended our study to the promoters of two additional members of the *Tc1/mariner* superfamily [*Sleeping Beauty* (SB) from fish and *Hsmar1* from human] and two additional LTR retrotransposons (*Tirant* and *ZAM*) from *D. melanogaster*. Furthermore, we included in this study the promoter of *hobo*, a member of the *hAT* superfamily (named after the *hobo*, *Activator* and *Tam3* founding elements [18]). The *hAT* superfamily represents an ideal outgroup to assess whether Class II elements, not related to the *Tc1/mariner* superfamily and able to undertake HTT [19–22], have blurry promoters that allow them to survive once transferred into distant genomes.

Our results suggest that the blurry promoter is a feature shared by the elements of the *Tc1/mariner* superfamily tested in this study, while the promoters of *hobo*, *Tirant* and *Zam* are usually functional within a limited range of genomic environments strictly related to the species of origin.

## Results

### Description of the sequences analyzed and outlined experimental strategy

*SB* and *Hsmar1* were used as representative elements to gain insights into promoter features of the *Tc1/mariner* superfamily. The promoter of the *hobo* element was chosen as an outgroup to Class II of transposons, whereas *Tirant* and *ZAM* were used as representative elements of the LTR retrotransposons belonging to the *Ty3-gypsy* superfamily. For the sake of comparison, the results obtained from the present work have been integrated with the results obtained in our recent study [16] concerning the promoters of *Bari1*, *Bari3* (two *Tc1*-like elements) and *copia* (a LTR retrotransposon of the *Ty1-copia* superfamily).

**Fig. 1:**
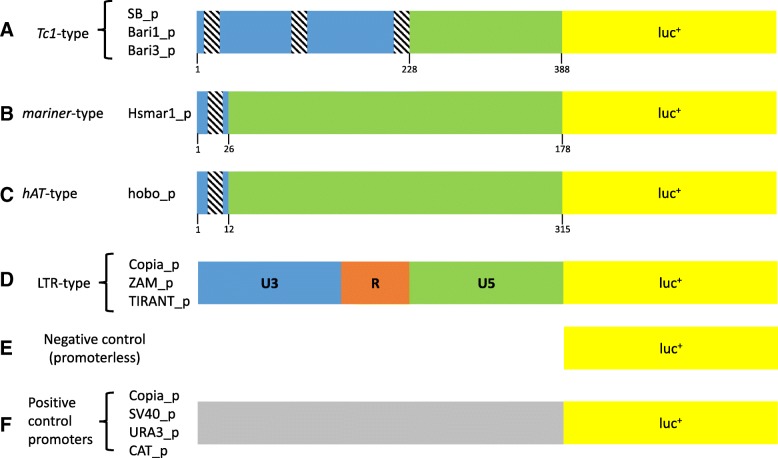
Overall scheme of the expression cassettes tested in this study. The tested sequences of Tc1-like*,* mariner-like and hobo elements (**a, b and c**) are divided into a blue region containing the transposase binding sites (depicted as dashed boxes) and sequences situated between the upstream TIRs and the transposase ORFs in these transposons (green region). These fragments are directly fused to a luciferase reporter gene (yellow). The LTR-retrotransposon sequences (**d**) are depicted with their canonical U3-R-U5 structure (more details are given in the main text). The organization of the negative (**e**) and positive (**f**) construct are also reported. Drawings are not in scale

The promoters of LTR retrotransposons usually maps within the 5′ LTR of the elements [23], whereas the promoters of *Tc1/mariner* elements are commonly located in the 5′ terminus of the element and, as previously suggested, within the intervening sequences between the TIR and the ATG codon of the transposase open reading frame (ORF) [16] [24]. We cloned fragments of the test TEs predicted to contain the promoters (Table 1) into reporter constructs. The 5′ terminal sequences of the *SB* transposon assayed in this study (hereafter SB_p) contain three binding sites, two essential and one dispensable, for the respective transposase [25] and also contains an intervening sequence, in which the transposon’s endogenous promoter has been previously mapped [24] (Fig. 1a). The *Hsmar1* 5′ terminal sequence tested (hereafter referred as Hsmar1_p), contains a single, 19-bp-long, transposase binding sequence mapping to the nucleotides 7–25 relative to the sequence tested [26] and a 152-bp-long flanking sequence (Fig. 1b). The *hobo* sequence tested spans the first 315 bp of the canonical element and consists of the 5′ TIR of 12 bp and an intervening sequence of 303 bp (Fig. 1c).

**Table 1:** Key features of the promoters analyzed in this study. The length of the tested fragment, the accession number of the reference element and its source organism, and the AT/GC content are listed for each element. References to previous works reporting the promoter characterization are also provided

Element	Cloned fragment (bp)	Reference element	Source Organism	AT%	GC%	Promoter characterization
*SB*	388	L48685.1 [27]	*T. albonubes*	63,66	36.34	[24], This study
*Bari1*	377	X67681.1 [28]	*D. melanogaster*	66,05	33.95	[16] [29]
*Bari3*	356	CH933806 [30]	*D. mojavensis*	65,23	34.77	[16] [31]
*Hsmar1*	178	U52077.1 [32]	*H. sapiens*	70,79	29,21	[33], This study
*hobo*	315	M69216.1 [18]	*D. melanogaster*	51,43	48,57	This study
*Tirant*	416	X93507.1 [34]	*D. melanogaster*	54,81	45,19	This study
*ZAM*	472	AJ000387.1 [35]	*D. melanogaster*	55,08	44,92	This study
*copia*	276	X02599.1 [36]	*D. melanogaster*	72,46	27,54	[16, 37]

The expression plasmids, carrying the luciferase reporter gene under the transcriptional control of the tested promoters, were either transiently (i.e. transfections into human HeLa cells and *Drosophila* S2R+ cells) or stably expressed (i.e. transformation in *S. cerevisiae* and *E. coli*) at the episomal level (see Material and Methods section). We used a promoter-less luciferase cassette as a negative control for background expression correction in each experiment (Fig. 1e). In addition, we used plasmids constitutively expressing luciferase under the control of strong species-specific promoters as positive controls, which also served as references to quantify the transposon-derived promoters’ activity (Fig. 1f).

### Activities of TE promoters in human cells

**Fig. 2:**
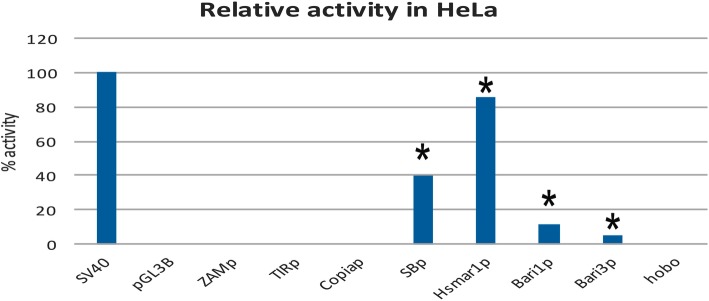
Promoter analysis in HeLa cells. Relative promoter activity, expressed as corrected mean RLU values, relative to the positive control (*SV40* promoter, set to 100) and to the promoter-less construct (set to 0). Promoters with activity significantly different from the promoter-less reporter cassette are marked with an asterisk. Actual values of each experiment set are shown in Additional file 5: Figure S1

We first tested the promoter activity of *SB* and *Hsmar1* in human HeLa cells (Fig. 2). As expected, and consistent with previously reported data [24, 33], SB_p and Hsmar1_p were able to drive reporter expression in cultured vertebrate cells (Fig. 2). In our hands, the SB_p has approximately 40% activity compared to the SV40 promoter. Similarly, Hsmar1_p is a strong promoter in HeLa cells (~ 80% activity when compared to SV40_p). As reported earlier [16], *Bari* promoters drive transcription of the reporter gene in HeLa cells. Conversely, the *hobo* promoter does not show any detectable activity in human cells. Likewise, the promoters of the retrotransposons *copia ZAM* and *Tirant* showed no detectable reporter activation over the background defined by the negative control (Fig. 2). These results suggest that the promoters isolated from the *Drosophila hobo, Tirant* and *Zam* elements do not support trans-Phylum transcription, i.e. in vertebrate cells.

### Transcriptional activity of transposon promoters in insect cells

**Fig. 3:**
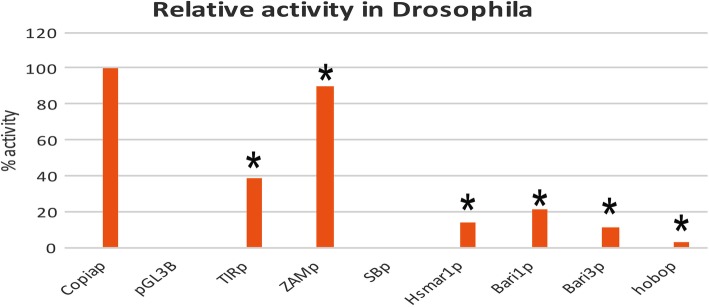
Promoter analysis in S2R+ cells. Relative promoter activity, expressed as corrected mean RLU values, relative to the positive control (*copia* promoter, set to 100) and to the promoter-less construct (set to 0). Promoters with activity significantly different from the promoter-less reporter cassette are marked with an asterisk. Actual values of each experiment set are shown in Additional file 6: Figure S2. The statistical significance of promoter activity against the promoter-less cassette is shown. **P < 0.005; ***P < 0.001

We next tested the ability of Hsmar1_p and SB_p to drive trans-Phylum transcription in insect cells by performing the luciferase assay in S2R+ *Drosophila* cells, using as a reference and positive control the pGL3B/copia expression vector. Whereas SB_p did not show an activity significantly different from the negative control, Hsmar1_p activity was roughly 14% compared to the *copia* promoter, suggesting that Hsmar1_p is an active promoter in *Drosophila* cells (Fig. 3). This result mirrors the ability of *Bari* promoters to drive trans-Phylum transcription human HeLa cells (Fig. 2).

The *hobo* promoter displayed significant, although weak, promoter activity in *Drosophila*, consistent with being an endogenous element of the fly genome [38].

Moreover, and consistently with our previous report, both *Bari* promoters were active in driving reporter gene expression in *Drosophila*. Finally, due to their retroviral nature, the promoters from *ZAM* and *Tirant* displayed an expected intense transcriptional activation of the reporter, as compared to the *copia* promoter (Fig. 3).

### Promoter analysis in yeast

**Fig. 4:**
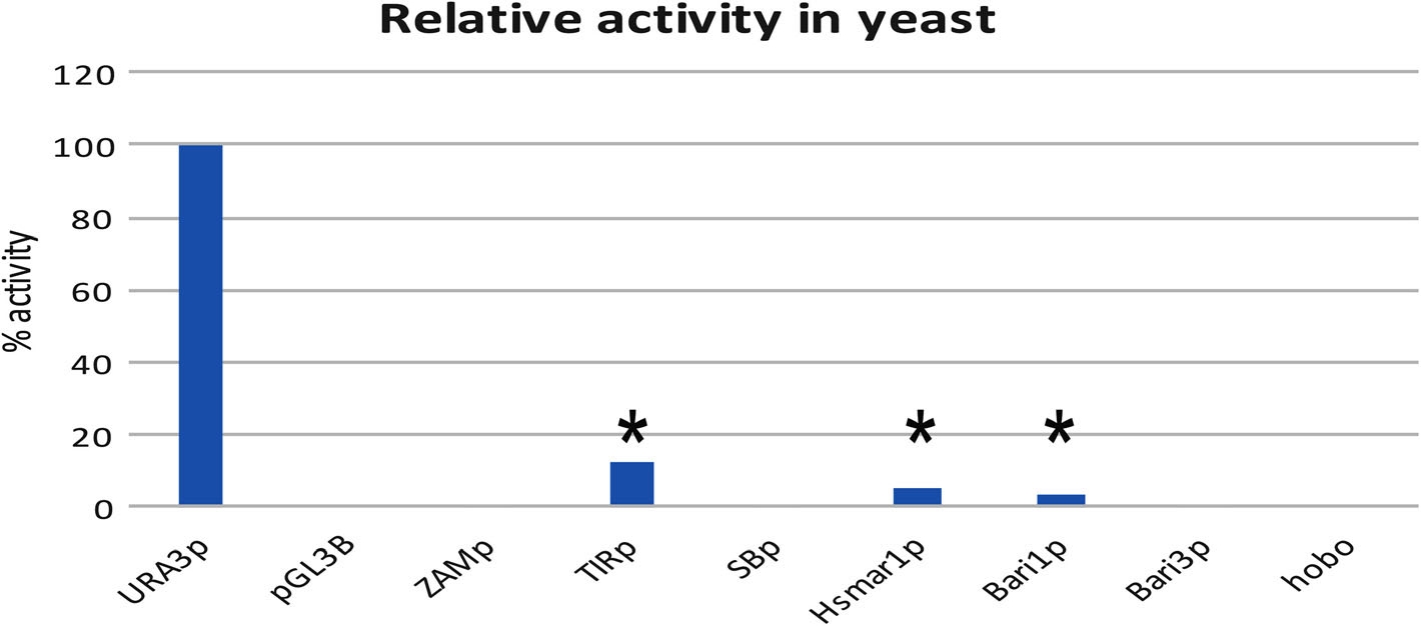
Promoter analysis in yeast cells. Relative promoter activity, expressed as corrected mean RLU values, relative to the positive control (URA3 promoter, set to 100) and to the promoter-less construct (set to 0). Promoters with activity significantly different from the promoter-less reporter cassette are marked with an asterisk. Actual values of each experiment set are shown in Additional file 7: Figure S3. The statistical significance of promoter activity against the promoter-less cassette is shown. *P < 0.05; **P < 0.005

In *S. cerevisiae* the activity of TE promoters was compared to the URA3 promoter and to the promoter-less construct. To this end, the same sequences tested in animal cells (i.e. human and insect cells) were sub-cloned into pFL39, a low-copy centromeric yeast vector that allowed an easy clonal analysis in *S. cerevisiae*. Interestingly, and similarly to what was previously found for Bari1_p, the activity of Hsmar1_p was about 4% of the strong *URA3* promoter, suggesting that it is a weak, but active promoter in yeast (Fig. 4). We also observed a promoter activity associated with the TIR_p sequence, at the level of 14% of the *URA3* promoter, a feature not shared by the *hobo* promoter and by the other LTR-derived promoters tested in this study (Fig. 4).

### Promoter analysis in bacteria

**Fig. 5:**
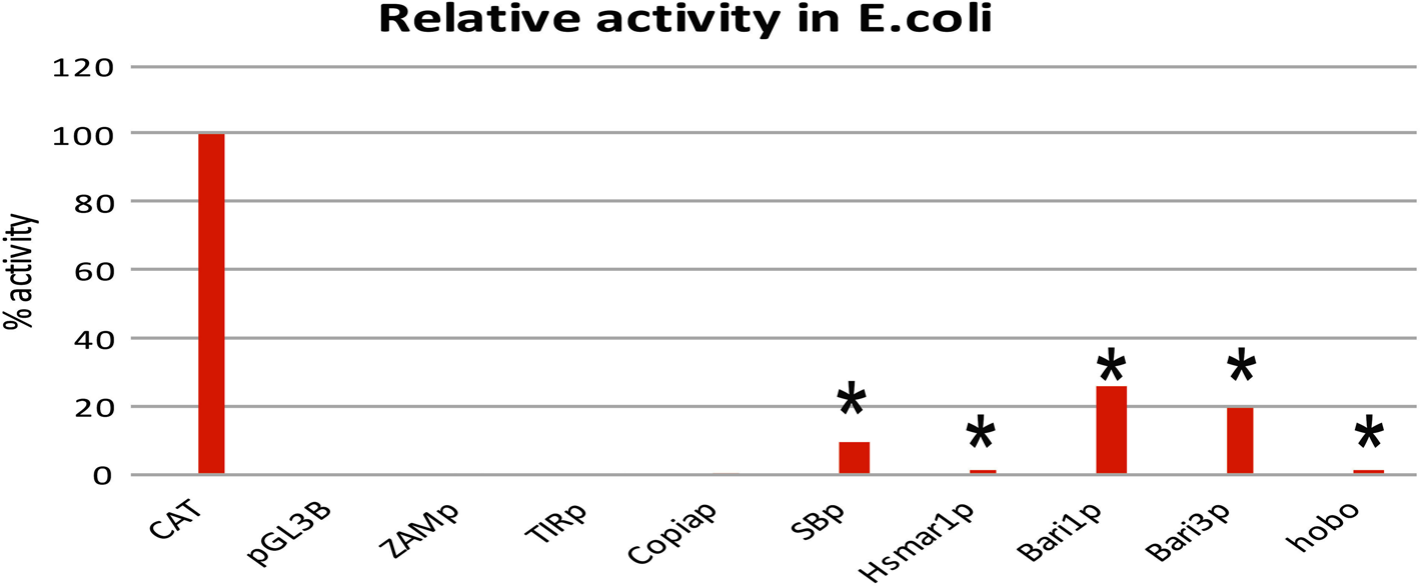
Promoter analysis in *E. coli* cells. Relative promoter activity, expressed as corrected mean RLU values, relative to the positive control (CAT promoter, set to 100) and to the promoter-less construct (set to 0). Promoters with activity significantly different from the promoter-less reporter cassette are marked with an asterisk. Actual values of each experiment set are shown in Additional file 8: Figure S4. The statistical significance of promoter activity against the promoter-less cassette is shown. ***P < 0.001

Luciferase assays conducted in *E. coli* showed that the activity of Hsmar1_p and hobo_p are very low when compared to the *CAT* promoter (0.15% and 0,42% respectively), but still significantly different from the promoter-less vector. Contrarily, SB_p displayed a strong activity, representing roughly 17% of the *CAT* promoter activity (Fig. 5). These results suggest that SB_p is more similar to the *Bari* promoters in *E. coli* (Fig. 5). No significant promoter activity has been observed to the tested sequences isolated from LTR-retrotransposons, which suggest the poor ability of these sequences to drive transcription in cells of a different domain of life.

### Eukaryotic core promoter motifs in test TEs

With the aim of identifying the core promoter sequences that could explain the observed behavior of the sequences tested in this study, we combined a bioinformatics approach with expressed sequence tags (ESTs) mapping and scientific literature data mining. We scanned the sequences of SB_p, Hsmar1_p, hobo_p, ZAM_p, TIR_p and Copia_p using matrices describing the TATA-box, the initiator element (InR) and the downstream promoter element (DPE), three DNA motifs commonly found in eukaryotic promoters. The results obtained using relaxed parameters (Additional file 1: Table S1, Additional file 2: Table S2 and Additional file 3: Table S3) show that it is not possible to identify all of the searched motifs in the *Tc1/mariner* elements. Indeed, SB_p lacks predictable Inr motifs, whereas two of the predicted TATA boxes and two of the predicted DPEs fall into the sequence previously described to contain the promoter [24].

The transcriptional start site (TSS) of *Hsmar1* can be inferred from a group of ESTs that arise from *Hsmar1* relics in the human genome (Additional File 4) that allow mapping of the TSS at position 130 in Hsmar1_p. The matrix-scan analysis performed on Hsmar1_p revealed two TATA boxes falling downstream the transposase binding site region, and mapping 56 and 28 bp upstream the inferred TSS. No Inr and DPE motifs can be detected using the matrix-scan approach.

The matrix-scan analysis of the *hobo* promoter reveals a high scoring TATA-box motif at position 105–116, which is in accordance with the TATA-box annotated in the GenBank record (M69216.1), and low-scoring Inr and DPE motifs at position 156–162 and 305–309 respectively. A CAAT box (position 49–52) is also annotated in the GenBank file. No transcripts arising from within the analyzed *hobo* sequence could be found in the database, in accordance with the reported low transcriptional activity of *hobo* in *D. melanogaster* [39].

The promoters of LTR-retrotransposons are usually located within specific LTR sub-regions. The LTRs of Ty3 and Ty1 elements usually contain three functional elements, namely U3 (Unique 3′ region), R (Repeated sequence) and U5 (Unique 5′ region) [40]. The R region starts invariably at the transcription start site and ends at the polyadenylation site. Therefore, the R sequence must contain the Inr motif and possibly the DPE motif, whereas the TATA box should be expected to lay in the U3 region. Previous studies have unveiled the U3-R-U5 structure of the *ZAM* element [35, 41, 42], which identified a 22-bp R region (nucleotides 326 to 347). A predicted TATA-box (Additional file 1: Table S1) maps at nucleotides 313–317, thus falling into the U3 region. The Inr of *ZAM* is defined by the TSS (also supported by a group of ESTs identified using BLASTn, see Additional File 4) across or near the R region start, although no sharp Inr can be predicted using our approach with the applied cutoff parameters (Additional file 2: Table S2). A potential DPE motif maps 24 bp downstream the Inr. The relative orientation and spacing of the three core promoter motifs are compatible, although sub-optimal, with the expected configuration of an ideal eukaryotic promoter [43].

To our knowledge, no data is available from the scientific literature concerning *Tirant* TSS. As a preliminary attempt to map the minimal promoter region of *Tirant*, we conducted a BLASTn search against the *D. melanogaster* ESTs database. As shown in Additional File 4, two different subsets of ESTs support two TSSs in the head and in embryos, thus predicting the possible U3-R boundary. One of the predicted TATA boxes and Inr motifs have compatible spacing (TATA-box at -28 bp, DPE at + 48 with respect to the Inr) with this predicted U3-R configuration.

It has been assessed by previous studies that the *copia* promoter lies within bases 70–276 [37]. Despite its wide use in expression vectors, little and contrasting information is available about the organization of its core-promoter motifs [37, 44, 45]. Our core-promoter motif prediction fits, in part, with the TATA box predicted at position 83–94, and the Inr (position 118–124) lying 6 bases upstream the Inr [45], and a DPE (position 179–183) outside the expected sequence spacing respect to the Inr motif.

Taken together, these results suggest that LTR-derived promoters and *hobo_p* might have a more structured organization compared to the promoters derived from *Tc1/mariner* elements, which more often have divergent, or sometimes lack, basal core-promoter elements, but still are able to drive transcription of a downstream gene.

## Discussion

TEs are special genomic components with an intrinsic ability to move within the same genome and across different genomes. This latter feature is more prominent for Class II transposons (i.e. DNA transposons), and particularly for *Tc1/mariner* elements [22]. However, the chance for survival of a TE upon HTT relies on several bottlenecks and on the possibility to express the transposase gene(s) [13].

The experimental procedure of transfection/transformation, routinely applied in many laboratories, mimics the naturally occurring HGT (and HTT) process, where the donor and receiving cells might be from different species. Thus, studying the activity of transcriptional regulatory sequences, using evolutionarily distant recipient cells in vitro*,* could allow us to infer how the tested sequences would determine the activation of gene expression after HGT. We have recently shown that the promoters isolated from *Bari1* and *Bari3*, two *Drosophila Tc1*-like transposons [46], are able to drive transcription of a downstream reporter gene in unrelated cellular environments, a feature not shared by the promoter of the *copia* retrotransposon. Based on this evidence, we have proposed that *Tc1*-like elements contain blurry promoters, i.e. AT-rich sequences containing weak core-promoter motifs [16].

In the present study, with the aim of validating the blurry promoter hypothesis and assess if this could be a feature shared with other TE families, we have analyzed the promoter region of three DNA transposons (*SB*, *Hsmar1* and *hobo*) and two LTR retrotransposons (*ZAM* and *Tirant*). Our analyses suggest that the promoter region of *SB* (a *Tc1*-like element) and *Hsmar1* (a *mariner*-like element) share structural and functional similarity with the promoter of *Bari* elements. First, a base usage analysis of the promoters from *Tc1/mariner* transposons studied reveals that they are all AT-rich in sequence. Conversely, the promoter of *hobo*, which belongs to the hAT superfamily, and the promoters of LTR-retrotransposons, with the exception of *copia* (72% AT), have an unbiased base content (Table 1). Second, the core-promoter search in the tested sequences suggests that the *Tc1/mariner* elements are less structured, might lack several core-promoter motifs and/or they might be unevenly spaced (Additional file 1: Table S1, Additional file 2: Table S2 and Additional file 3: Table S3). Third, the promoter of *Tc1/mariner* elements is the ability to activate gene transcription in distant hosts, a distinctive feature of the transposon’s “blurry” promoters. Blurry promoters could predispose certain TEs to be more successful in HTT than other coding and noncoding DNA sequences [21], thereby contributing to the success of TEs in establishing a foothold in a new genome upon a HTT event. Here, we show that this feature is not distinctive of the two *Bari* transposon promoters, nor limited to transposons of the *Drosophila* genus, but it can be extended at least to other members of the *Tc1/mariner* superfamily.

*SB* and *Hsmar1* are two inactive transposable elements found in vertebrates, whose transposase genes have been reconstructed through in vitro site directed mutagenesis [33, 47]. Thousands of *SB* and *Hsmar1* relics can be found in extant salmonid fish and primate genomes respectively. For both transposons, the terminal sequences were found to be the most conserved in sequence, i.e. less divergent due to the accumulation of disrupting mutations. Indeed, the SB_p sequence used in this study was derived from a *Tc1-*like element isolated from the White Cloud Mountain fish, *Tanichthys albonubes* [47], whereas the Hsmar1_p sequence is part of a consensus assembled from the human genome [33]. The results obtained in this study clearly show that similarly to what was observed for the promoter of *Bari* elements, SB_p and Hsmar1_p are able to drive transcription in distant genomes.

Unlike SB_p and Hsmar1_p, neither the LTR-derived promoters nor hobo_p displayed significant activity in HeLa cells. While it would be expected that vertebrate promoters could be active in a vertebrate non-resident cellular environment, it should be noted that primates and fishes (*Chondrichthyes*) shared their last common ancestor more than 460 Mya (source http://www.timetree.org/). Despite their ancient relationship, the transcriptional machinery in fishes and humans are apparently able to cross-recognize *cis*-acting sequences, suggesting that some TFs and TFBSs are interchangeable in these two lineages. This observation is also supported by several studies in which fish promoters were tested in human cells [48, 49].

The transfection of SB_p and Hsmar1_p into *Drosophila* cells (Fig. 3) mimics an inter-phyla HTT event, from vertebrates to insects. Our results show that, unlike SB_p, Hsmar1_p is an active promoter in *Drosophila* and its activity is comparable to that of Bari3_p. Although we could detect a promoter activity significantly different from the promoter-less control the hobo_p promoter is a weak promoter in *Drosophila* (Fig. 2). Previous works have highlighted that *hobo* transcriptional activity can only be detected in the germline, with very poor (or absent) somatic expression in *Drosophila* [39], suggesting strong transcriptional regulation of transposase expression. The S2R+ cell line used in this study is indeed of somatic origin [50] thus explaining the observed low promoter activity in our assay.

Even more strikingly, we have observed promoter activity of SB_p in bacteria and of Hsmar1_p in yeast (Fig. 4). Transformation into yeast and bacteria of vertebrate sequences could be seen as laboratory recapitulations of inter-Kingdom HTT events (i.e. HTT events involving animals and fungi) and inter-Domain HTT events, (i.e. HTT event involving a eukaryote and a prokaryote). While promoters from *Tc1/mariner* like transposons are generally active upon inter-Kingdom or inter-Domain transfer, the LTR-derived promoters appear to be less prone to support transcription in distantly related hosts (Figs. 4 and 5). However, some of the non-*Tc1/mariner* elements tested in this study are also able to drive gene expression outside their native host genetic environment (Figs. 4 and 5). This observation implies that many other types of TEs could be able to activate transcription of their transposition-related enzymes when they move from the species of origin to an unrelated one, but in a more narrow range of species if compared to *Tc1/mariner* elements. In conclusion, the results presented in this study involving the promoters from TEs of different types (i.e. 4 *Tc1/mariner*, 1 hAT and 3 LTR-retrotransposons elements), suggest that *Tc1/mariner* elements are more promiscuous in activating transcription in distantly related hosts, a feature that could be linked to their enhanced success in the HTT process [21, 22].

### Possible impact of HGT of active promoters on genes, genomes and adaptation

Despite being inactive as transposons, several thousands of *SB* and *Hsmar1* relics, most of which contain the promoter sequences, can be found in the respective host genomes, suggesting possible impact on gene regulation in their native hosts. For example, transposon insertions can affect host gene expression by juxtaposition of their transcriptional regulatory sequences to endogenous transcription units, thereby overriding their physiological transcriptional program. *D. melanogaster* provides significant examples of transcriptional perturbation of host genes [51–55] due to potent TE-related *cis*-acting regulatory sequences, such as enhancers, silencers or insulators, mainly associated with LTR-retrotransposons [56–61]. Our results suggest that promoters of the analyzed TEs act as weak transcriptional activators, especially when they move to distant hosts. For instance, the promoters of *Hsmar1* and *hobo* have a very low, although statistically significant, activity in *E. coli*. This could still have a biological significance, especially in the absence of additional repressive mechanisms that acted on the TE in the species of origin, leading to a successful invasion of the new genome.

In natural ecosystems, the relationships between different lineages have been established during evolution. Environmental perturbations could be seen as genetic stressors by the community members, which would react promptly especially with the acquisition of novel traits allowing them to persist in the same environment or to colonize new environments. The role of HGT/HTT in the determination of gene flow in ecological niches is well recognized [62]. In this context, genome colonization by TEs could be a critical contributor to eliciting complex genetic changes occurring in ecological niches of special interest, such as intestine and soil. Investigations of TE-associated promoters will help understanding the potential invasiveness of the TE in new genomes and the potential ability to rewire pre-existing transcriptional circuitries, leading to adaptation in a given eco-niche.

### Potential biotechnological application of TE- related promoters

To date, few promoters of viral origin are known to drive transcription in multiple hosts. As an example the *35S* promoter of the *CaMV* virus, a strong constitutive plant promoter [63], displays its activity in virtually all organisms, from higher vertebrates to prokaryotes [64] [65]. Similarly, although not so strikingly, the promoter of the crustacean *IHHNV* virus, is able to drive the transcription of reporter genes in insects and fish cells [66]. Given the non-viral origin of TEs, these sequences, especially Class II transposons, have been recently highly regarded in the development of biotechnological tools, especially in the field of gene therapy [67]. Moreover, some TE-related regulatory sequences (e.g. the *copia* promoter) have been used to develop commercially available in vitro gene expression tools.

The observation that the promoters of some transposon families display transcriptional activation of downstream genes in multiple cellular hosts enables the development of expression vectors based on blurry promoters. Enabling ectopic expression in many cellular model systems using a single recombinant construct could be useful in basic research to accelerate industrial production processes. The weakness of the promoters described here could limit their use in such applications. However, this could be a desirable feature to express genes closer to their physiological levels. To better assess the strength of TE-derived promoters a comparison with weaker promoters will be needed. Further investigations in the field of TEs and mutagenesis of preliminary characterized TE- related promoters could hopefully lead to a wider application of TE-derived regulatory sequences for the development of new biotechnological tools with a broader range of transcriptional activation in multiple hosts.

## Conclusion

This study demonstrates that, similar to the *Bari* elements isolated from *Drosophila*, the *SB* (from fish) and *Hsmar1* (human) transposons contain transcriptionally promiscuous “blurry” promoters. The blurry promoter could be a feature widely shared among the members of the *Tc1*/*mariner* superfamily.

The results obtained in this work confirm our previous hypothesis that the promoters of TEs from the *Tc1*/*mariner* superfamily could contribute to the success and spread of these mobile elements and might represent one of the keys towards the full understanding of the complex phenomenon of HTT.

Given the high number of annotated and characterized TEs, we believe that other elements could also carry promiscuous promoters. Continuing effort put into the study of TE’s regulatory sequences, would enable the discovery of additional peculiar features that could be used in biotechnological applications and would allow a significant advancement in the field of TE biology.

## Materials and methods

S2R+ cells were passaged in Schneider’s Insect Medium supplemented with 10% FBS, 1% penicillin/streptomycin, and maintained at 25 °C. HeLa cells were maintained at 37 °C with 5% CO2 in Dulbecco’s Minimum Essential Medium supplemented with 10% FBS, 200 mM glutamine, 1% penicillin/streptomycin. *Saccharomyces cerevisiae*, strain BMA64-1A (MATa leu2–3112 his3–11,15 trp1∆ ade2–1 ura3–1), was cultured on Synthetic Complete medium [68] supplemented with 2% glucose as carbon source. *Escherichia coli* cultures, strain DH5alpha, were grown on selective LB medium supplemented with ampicillin.

The transposable elements’ fragments analyzed in this study have been amplified using the primers listed in Table 2 and cloned into the pGL3B vector. The promoter of *hobo*, *ZAM* and *Tirant* were amplified from the *D. melanogaster* reference strain genomic DNA (y^1^; cn^1^, bw^1^, sp^1^). The entire expression cassettes (i.e. promoter-luc + SV40 terminator) were cut out from the pGL3B vector and sub-cloned into pFL39 vector [69] using either KpnI and BamHI or the SacI and SalI restriction sites. The pFL39 vector is a centromeric yeast plasmid that allows selection of stably transformed clones on selective media lacking tryptophan. The constructs containing *Bari1* and *Bari3* promoters, as well as the control promoters used in this study (namely Copia_p, URA3_p, and CAT_p), have been described in [16]. Sequence verification of all the cloned fragments was performed at the BMR-Genomics (Padova, Italy) using the Luc278_rev sequencing primer.

**Table 2:** List of primers used in this study

SB_f	CTCGAGCAGTTGAAGTCGGAAGTTTACATACACTTAGGTTGGAG
SB_r	CCATGGATGTTTTTGGCGTCTTCCATGATGTCAAGCAAAGAGGCACTG
Hsmar1_f	CTCGAGTTAGGTTGGTGCAAAAGTAATTGC
Hsmar1_r	CCATGGAGTCTAAAATAAACATAAAATAAACA
Tirant_LTR_f	CTCGAGGGAGTTACCACCCCACCCCCTA
Tirant_LTR_r	AGATCTCAGTTAAGTCCGTGATCGAGGGT
ZAM_LTR_f	CTCGAGTACCGACCCATCGGTACCATAC
ZAM_LTR_r	CCATGGGCGCAGTTACCTCCGGGGAGTCT
hobo_prom_UP	GATCCTCGAGCAGAGAACTGCAAGGGTGGCACT
hobo_prom_LOW	GATCCCATGGTTGACTCGACTACCTACGAGA
Luc278_rev	GCCCAACACCGGCATAAAGAATT

Transfections were performed in 6-well plates using TransIt LT1 (Mirus Bio, Madison, WI), and 1 μg of the appropriate plasmid or co-transfected with the Renilla luciferase construct (pRL-SV40; Promega, Madison, WI, USA), according to the manufacturer instructions. Yeast transformation was performed using the TRAFO methods described in [70]. Bacteria transformation was performed using standard transformation protocol and chemically competent cells [71]. Luciferase activities measurements were recorded on GLOMAX 20/20 Luminometer (Promega, Madison, WI, USA) 24 h post-transfection. *S. cerevisiae* and *E. coli* transformants were assayed in the log-phase. We recorded three independent luciferase activity measurements per sample and the average value taken as sample measure. Experiments were made at least in triplicates.

RLUs obtained for the tested promoters, were corrected for the RLU value of the respective negative control, and normalized to the total protein content measured using the Bradford Assay or to the Renilla luciferase measure.

The normalized luciferase activity of positive controls, were arbitrarily set to 100, whereas the negative controls were set to zero in each experiment. T-student test was carried out to assess the statistical significance of the differences observed between the transposons’ promoter tested and the respective promoter-less construct (H0: normalized RLU measurement is not different between test promoter and negative control promoter).

Positional weight matrices used to map the TATA-box, Inr (Initiator Element) and DPE (Downstream Promoter Element) core-promoter motifs were retrieved at YAPP Eukaryotic Core Promoter Predictor (www.bioinformatics.org/yapp/cgi-bin/yapp.cgi; last accessed June 2018). Matrix scan analysis was performed using Regulatory Sequence Analysis Tools (RSAT) (http://pedagogix-tagc.univ-mrs.fr/rsat/RSAT_portal.html; last accessed July 2018) [72]).

BLAST searches were performed at the NCBI (https://blast.ncbi.nlm.nih.gov/Blast.cgi) against appropriate databases.

## Additional files

Additional file 1: **Table S1.** TATA box motif prediction. Pval cutoff 10exp-2; origin = START; only direct strand results are shown (DOCX 180 kb)

Additional file 2: **Table S2.** Inr motif prediction. Pval cutoff 10exp-2; origin = START; only direct strand results are shown (DOCX 115 kb)

Additional file 3: **Table S3.** DPE motif prediction. Pval cutoff 10exp-2; origin = START; only direct strand results are shown (DOCX 151 kb)

Additional File 4: Accessions of cDNA/ESTs supporting TSS predicted in the sequences tested (TXT 643 bytes)

Additional file 5: **Figure S1.** Luciferase-promoter assay in HeLa cells. Individual promoter-luciferase assay results in HeLa cells. Top and bottom whiskers: maximum and minimum values of each samples respectively. Top and bottom of boxes: 75th and 25th percentile of the samples respectively. Line through the boxes: median of each sample. X markers: mean of each samples. The statistical significance of promoter activity against the promoter-less cassette is shown. *P < 0.05; **P < 0.005; ***P < 0.001 (PDF 168 kb)

Additional file 6: **Figure S2.** Luciferase-promoter assay in S2R+ cells. Individual promoter-luciferase assay results in S2R+ cells of *D. melanogaster*. Top and bottom whiskers: maximum and minimum values of each samples respectively. Top and bottom of boxes: 75th and 25th percentile of the samples respectively. Line through the boxes: median of each sample. X markers: mean of each samples. The statistical significance of promoter activity against the promoter-less cassette is shown. **P < 0.005; ***P < 0.001 (PDF 174 kb)

Additional file 7: **Figure S3.** Luciferase-promoter assay in *S. cerevisiae* cells (BMA64-1A). Individual promoter-luciferase assay results in yeast. Top and bottom whiskers: maximum and minimum values of each samples respectively. Top and bottom of boxes: 75th and 25th percentile of the samples respectively. Line through the boxes: median of each sample. X markers: mean of each samples. The statistical significance of promoter activity against the promoter-less cassette is shown. *P < 0.05; **P < 0.005 (PDF 216 kb)

Additional file 8: **Figure S4.** Luciferase-promoter assay in bacteria (DH5alpha cells). Individual promoter-luciferase assay results in DH5alpha cells. Top and bottom whiskers: maximum and minimum values of each samples respectively. Top and bottom of boxes: 75th and 25th percentile of the samples respectively. Line through the boxes: median of each sample. X markers: mean of each samples. The statistical significance of promoter activity against the promoter-less cassette is shown. ***P < 0.001 (PDF 154 kb)

## Abbreviation

EST: Expressed Sequence Tags
HGT: Horizontal Gene Transfer
HTT: Horizontal Transposon Transfer
LTR: Long Terminal Repeat
ORF: Open Reading Frame
R: Repeated sequence
TEs: Transposable Elements
TF: Transcription Factor
TFBS: Transcription Factor Binding Site
TIRs: Terminal Inverted Repeats
TSS: Transcriptional Start Site
U3: Unique 3′ region
U5: Unique 5′ region
