# Supplementary material for: Transcriptionally promiscuous “blurry” promoters in Tc1/mariner transposons allow transcription in distantly related genomes
Source: Mob DNA. 2019 Apr 3;10:13. doi: 10.1186/s13100-019-0155-6 (PMC6446368; doi:10.1186/s13100-019-0155-6)
Supplement: Supplementary file 1 — Table S1. TATA box motif prediction. Pval cutoff 10exp-2; origin = START; only direct strand results are shown (DOCX 180 kb) [file 13100_2019_155_MOESM1_ESM.docx]

| **seq_id** | **ft_type** | **ft_name** | **start** | **end** | **sequence** | **weight** | **Pval** | **ln_Pval** | **sig** | **rank** |
| --- | --- | --- | --- | --- | --- | --- | --- | --- | --- | --- |
| SB | limit | START_END | 1 | 388 | . | 0 | 0 | 0 | 0 |  |
| **SB** | **site** | **matrix-scan_2018-07-28** | **39** | **50** | **TCATTAAAACTC** | **2.1** | **6.3e-03** | **-5.065** | **2.199** | **1** |
| SB | site | matrix-scan_2018-07-28 | 277 | 288 | GGCTTTAGAAGC | 1.5 | 8.4e-03 | -4.777 | 2.074 | 2 |
| SB | site | matrix-scan_2018-07-28 | 278 | 289 | GCTTTAGAAGCT | 1.4 | 8.9e-03 | -4.725 | 2.052 | 3 |
| SB | site | matrix-scan_2018-07-28 | 38 | 49 | GTCATTAAAACT | 1.1 | 1.0e-02 | -4.586 | 1.992 | 4 |
| SB | site | matrix-scan_2018-07-28 | 238 | 249 | CCTTTAAACAGC | 1.1 | 1.0e-02 | -4.586 | 1.992 | 5 |
| hsmar | limit | START_END | 1 | 178 | . | 0 | 0 | 0 | 0 |  |
| **hsmar** | **site** | **matrix-scan_2018-07-28** | **72** | **83** | **TAAATAAATGTG** | **4.5** | **1.5e-03** | **-6.482** | **2.815** | **1** |
| hsmar | site | matrix-scan_2018-07-28 | 98 | 109 | CATTTTAATGCG | 2.9 | 4.2e-03 | -5.475 | 2.378 | 2 |
| **hsmar** | **site** | **matrix-scan_2018-07-28** | **68** | **79** | **TTCTTAAATAAA** | **1.4** | **8.9e-03** | **-4.725** | **2.052** | **3** |
| hobo | limit | START_END | 1 | 315 | . | 0 | 0 | 0 | 0 |  |
| hobo | site | matrix-scan_2019-02-03 | 105 | 116 | AGTATAAATCTG | 6.7 | 2.2e-04 | -8.425 | 3.659 | 1 |
| hobo | site | matrix-scan_2019-02-03 | 197 | 208 | CCTAAAAAGGGA | 2.2 | 4.7e-03 | -5.352 | 2.324 | 2 |
| hobo | site | matrix-scan_2019-02-03 | 195 | 206 | TGCCTAAAAAGG | 1.5 | 6.6e-03 | -5.026 | 2.183 | 3 |
| copia | limit | START_END | 1 | 276 | . | 0 | 0 | 0 | 0 |  |
| copia | site | matrix-scan_2018-07-28 | 83 | 94 | GCCATAAAACAT | 3.8 | 2.5e-03 | -6.006 | 2.608 | 1 |
| copia | site | matrix-scan_2018-07-28 | 195 | 206 | AATATAAATCAT | 3.4 | 3.2e-03 | -5.760 | 2.502 | 2 |
| copia | site | matrix-scan_2018-07-28 | 254 | 265 | AATATAAATTAT | 3.3 | 3.3e-03 | -5.708 | 2.479 | 3 |
| copia | site | matrix-scan_2018-07-28 | 261 | 272 | ATTATAAATTAC | 3.3 | 3.3e-03 | -5.708 | 2.479 | 4 |
| copia | site | matrix-scan_2018-07-28 | 59 | 70 | GATATGAATGGC | 2.9 | 4.2e-03 | -5.475 | 2.378 | 5 |
| copia | site | matrix-scan_2018-07-28 | 193 | 204 | AAAATATAAATC | 2.7 | 4.6e-03 | -5.374 | 2.334 | 6 |
| copia | site | matrix-scan_2018-07-28 | 202 | 213 | ATCATAAAACTC | 2.6 | 4.9e-03 | -5.321 | 2.311 | 7 |
| copia | site | matrix-scan_2018-07-28 | 252 | 263 | GAAATATAAATT | 2.0 | 6.6e-03 | -5.016 | 2.179 | 8 |
| copia | site | matrix-scan_2018-07-28 | 48 | 59 | ACTTTATATTTG | 1.8 | 7.3e-03 | -4.914 | 2.134 | 9 |
| copia | site | matrix-scan_2018-07-28 | 239 | 250 | TTTATTAAGAAA | 1.3 | 9.3e-03 | -4.683 | 2.034 | 10 |
| zam | limit | START_END | 1 | 472 | . | 0 | 0 | 0 | 0 |  |
| zam | site | matrix-scan_2018-07-28 | 311 | 322 | AGAATAAAGACG | 5.6 | 6.3e-04 | -7.368 | 3.200 | 1 |
| zam | site | matrix-scan_2018-07-28 | 297 | 308 | CCCATTTAAGAC | 2.6 | 4.9e-03 | -5.321 | 2.311 | 2 |
| zam | site | matrix-scan_2018-07-28 | 299 | 310 | CATTTAAGACGA | 1.8 | 7.3e-03 | -4.914 | 2.134 | 3 |
| zam | site | matrix-scan_2018-07-28 | 206 | 217 | CGTATAAACATA | 1.4 | 8.9e-03 | -4.725 | 2.052 | 4 |
| zam | site | matrix-scan_2018-07-28 | 196 | 207 | CATATACATACG | 1.2 | 9.7e-03 | -4.636 | 2.014 | 5 |
| TIRANT | limit | START_END | 1 | 416 | . | 0 | 0 | 0 | 0 |  |
| TIRANT | site | matrix-scan_2018-07-28 | 222 | 233 | TGTATAAGACGA | 5.4 | 7.5e-04 | -7.195 | 3.125 | 1 |
| TIRANT | site | matrix-scan_2018-07-28 | 348 | 359 | AACTTAAAAACC | 3.1 | 3.7e-03 | -5.593 | 2.429 | 2 |
| TIRANT | site | matrix-scan_2018-07-28 | 220 | 231 | TATGTATAAGAC | 1.1 | 1.0e-02 | -4.586 | 1.992 | 3 |

SUPPLEMENTARY TABLE 1. TATA BOX MOTIF PREDICTION. Pval cutoff 10exp-2; origin=START; only direct strand results are shown
