# Supplementary material for: Transcriptionally promiscuous “blurry” promoters in Tc1/mariner transposons allow transcription in distantly related genomes
Source: Mob DNA. 2019 Apr 3;10:13. doi: 10.1186/s13100-019-0155-6 (PMC6446368; doi:10.1186/s13100-019-0155-6)
Supplement: Supplementary file 2 — Table S2. Inr motif prediction. Pval cutoff 10exp-2; origin = START; only direct strand results are shown (DOCX 115 kb) [file 13100_2019_155_MOESM2_ESM.docx]

| **seq_id** | **ft_type** | **ft_name** | **strand** | **start** | **end** | **sequence** | **weight** | **Pval** | **ln_Pval** | **sig** | **rank** |
| --- | --- | --- | --- | --- | --- | --- | --- | --- | --- | --- | --- |
| SB | limit | START_END | D | 1 | 388 | . | 0 | 0 | 0 | 0 |  |
| SB | site | matrix-scan_2018-07-30 | D | 318 | 324 | TCAATTG | 3.5 | 8.9e-03 | -4.719 | 2.049 | 1 |
| hsmar | limit | START_END | D | 1 | 178 | . | 0 | 0 | 0 | 0 |  |
| hobo | limit | START_END | D | 1 | 315 | . | 0 | 0 | 0 | 0 |  |
| hobo | site | matrix-scan_2019-02-03 | D | 30 | 36 | CCACTCG | 5.1 | 1.3e-03 | -6.663 | 2.894 | 1 |
| hobo | site | matrix-scan_2019-02-03 | D | 67 | 73 | CTACTCG | 4.5 | 3.5e-03 | -5.652 | 2.455 | 2 |
| hobo | site | matrix-scan_2019-02-03 | D | 156 | 162 | TCAATAC | 4.4 | 3.8e-03 | -5.575 | 2.421 | 3 |
| hobo | site | matrix-scan_2019-02-03 | D | 39 | 45 | TCACACC | 4.3 | 4.1e-03 | -5.496 | 2.387 | 4 |
| copia | limit | START_END | D | 1 | 276 | . | 0 | 0 | 0 | 0 |  |
| copia | site | matrix-scan_2018-07-30 | D | 109 | 115 | CCACTCT | 5.1 | 8.4e-04 | -7.080 | 3.075 | 1 |
| copia | site | matrix-scan_2018-07-30 | D | 70 | 76 | CCACACC | 4.1 | 4.3e-03 | -5.447 | 2.366 | 2 |
| copia | site | matrix-scan_2018-07-30 | D | 118 | 124 | TTATTCC | 3.6 | 7.9e-03 | -4.847 | 2.105 | 3 |
| zam | limit | START_END | D | 1 | 472 | . | 0 | 0 | 0 | 0 |  |
| zam | site | matrix-scan_2018-07-30 | D | 156 | 162 | CCACATC | 4.4 | 2.7e-03 | -5.905 | 2.565 | 1 |
| zam | site | matrix-scan_2018-07-30 | D | 124 | 130 | TCAGACG | 3.9 | 5.6e-03 | -5.185 | 2.252 | 2 |
| TIRANT | limit | START_END | D | 1 | 416 | . | 0 | 0 | 0 | 0 |  |
| TIRANT | site | matrix-scan_2018-07-30 | D | 300 | 306 | TCAGTCG | 5.4 | 4.6e-04 | -7.687 | 3.339 | 1 |
| TIRANT | site | matrix-scan_2018-07-30 | D | 253 | 259 | CCACTCT | 5.1 | 8.4e-04 | -7.080 | 3.075 | 2 |
| TIRANT | site | matrix-scan_2018-07-30 | D | 376 | 382 | TCATATC | 4.2 | 3.6e-03 | -5.624 | 2.443 | 3 |

SUPPLEMENTARY TABLE 2. INR MOTIF PREDICTION. Pval cutoff 10exp-2; origin=START; only direct strand results are shown
