# Supplementary material for: Transcriptionally promiscuous “blurry” promoters in Tc1/mariner transposons allow transcription in distantly related genomes
Source: Mob DNA. 2019 Apr 3;10:13. doi: 10.1186/s13100-019-0155-6 (PMC6446368; doi:10.1186/s13100-019-0155-6)
Supplement: Supplementary file 3 — Table S3. DPE motif prediction. Pval cutoff 10exp-2; origin = START; only direct strand results are shown (DOCX 151 kb) [file 13100_2019_155_MOESM3_ESM.docx]

| **seq_id** | **ft_type** | **ft_name** | **strand** | **start** | **end** | **sequence** | **weight** | **Pval** | **ln_Pval** | **sig** | **rank** |
| --- | --- | --- | --- | --- | --- | --- | --- | --- | --- | --- | --- |
| SB | limit | START_END | D | 1 | 388 | . | 0 | 0 | 0 | 0 |  |
| SB | site | matrix-scan_2018-07-30 | D | 8 | 12 | AGTCG | 5.1 | 8.7e-04 | -7.053 | 3.063 | 1 |
| SB | site | matrix-scan_2018-07-30 | D | 338 | 342 | GGATG | 4.9 | 1.9e-03 | -6.284 | 2.729 | 2 |
| SB | site | matrix-scan_2018-07-30 | D | 2 | 6 | AGTTG | 3.9 | 7.9e-03 | -4.846 | 2.105 | 3 |
| SB | site | matrix-scan_2018-07-30 | D | 31 | 35 | AGTTG | 3.9 | 7.9e-03 | -4.846 | 2.105 | 4 |
| SB | site | matrix-scan_2018-07-30 | D | 227 | 231 | AGTTG | 3.9 | 7.9e-03 | -4.846 | 2.105 | 5 |
| SB | site | matrix-scan_2018-07-30 | D | 205 | 209 | GGTCA | 3.8 | 9.0e-03 | -4.710 | 2.045 | 6 |
| hsmar | limit | START_END | D | 1 | 178 | . | 0 | 0 | 0 | 0 |  |
| hsmar | site | matrix-scan_2018-07-30 | D | 4 | 8 | GGTTG | 4.3 | 4.4e-03 | -5.428 | 2.358 | 1 |
| hobo | limit | START_END | D | 1 | 315 | . | 0 | 0 | 0 | 0 |  |
| hobo | site | matrix-scan_2019-02-03 | D | 206 | 210 | GGATG | 4.6 | 1.8e-03 | -6.335 | 2.751 | 1 |
| hobo | site | matrix-scan_2019-02-03 | D | 305 | 309 | AGTCG | 4.3 | 5.4e-03 | -5.227 | 2.270 | 2 |
| copia | limit | START_END | D | 1 | 276 | . | 0 | 0 | 0 | 0 |  |
| copia | site | matrix-scan_2018-07-30 | D | 159 | 163 | GGTCG | 5.5 | 2.0e-04 | -8.531 | 3.705 | 1 |
| copia | site | matrix-scan_2018-07-30 | D | 179 | 183 | AGTTG | 3.9 | 7.9e-03 | -4.846 | 2.105 | 2 |
| zam | limit | START_END | D | 1 | 472 | . | 0 | 0 | 0 | 0 |  |
| zam | site | matrix-scan_2018-07-30 | D | 328 | 332 | AGTCG | 5.1 | 8.7e-04 | -7.053 | 3.063 | 1 |
| zam | site | matrix-scan_2018-07-30 | D | 126 | 130 | AGACG | 4.9 | 1.9e-03 | -6.284 | 2.729 | 2 |
| zam | site | matrix-scan_2018-07-30 | D | 305 | 309 | AGACG | 4.9 | 1.9e-03 | -6.284 | 2.729 | 3 |
| zam | site | matrix-scan_2018-07-30 | D | 318 | 322 | AGACG | 4.9 | 1.9e-03 | -6.284 | 2.729 | 4 |
| zam | site | matrix-scan_2018-07-30 | D | 355 | 359 | AGACG | 4.9 | 1.9e-03 | -6.284 | 2.729 | 5 |
| zam | site | matrix-scan_2018-07-30 | D | 388 | 392 | AGACG | 4.9 | 1.9e-03 | -6.284 | 2.729 | 6 |
| zam | site | matrix-scan_2018-07-30 | D | 409 | 413 | GGACC | 4.4 | 3.8e-03 | -5.564 | 2.416 | 7 |
| zam | site | matrix-scan_2018-07-30 | D | 97 | 101 | AGTTG | 3.9 | 7.9e-03 | -4.846 | 2.105 | 8 |
| zam | site | matrix-scan_2018-07-30 | D | 122 | 126 | GGTCA | 3.8 | 9.0e-03 | -4.710 | 2.045 | 9 |
| TIRANT | limit | START_END | D | 1 | 416 | . | 0 | 0 | 0 | 0 |  |
| TIRANT | site | matrix-scan_2018-07-30 | D | 134 | 138 | AGTCG | 5.1 | 8.7e-04 | -7.053 | 3.063 | 1 |
| TIRANT | site | matrix-scan_2018-07-30 | D | 177 | 181 | AGTCG | 5.1 | 8.7e-04 | -7.053 | 3.063 | 2 |
| TIRANT | site | matrix-scan_2018-07-30 | D | 302 | 306 | AGTCG | 5.1 | 8.7e-04 | -7.053 | 3.063 | 3 |
| TIRANT | site | matrix-scan_2018-07-30 | D | 66 | 70 | AGACG | 4.9 | 1.9e-03 | -6.284 | 2.729 | 4 |
| TIRANT | site | matrix-scan_2018-07-30 | D | 228 | 232 | AGACG | 4.9 | 1.9e-03 | -6.284 | 2.729 | 5 |

SUPPLEMENTARY TABLE 3. DPE MOTIF PREDICTION. Pval cutoff 10exp-2; origin=START; only direct strand results are shown.
