# Supplementary figures and images for: Transcriptionally promiscuous “blurry” promoters in Tc1/mariner transposons allow transcription in distantly related genomes
Source: Mob DNA. 2019 Apr 3;10:13. doi: 10.1186/s13100-019-0155-6 (PMC6446368; doi:10.1186/s13100-019-0155-6)

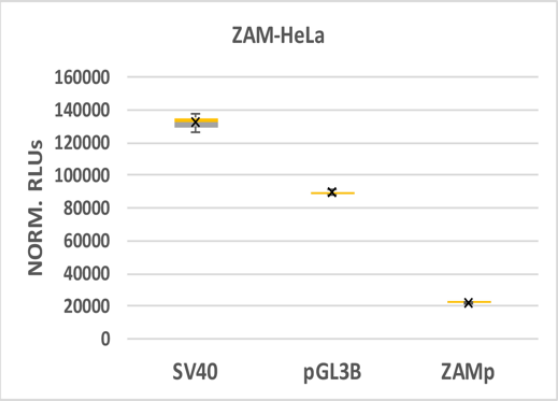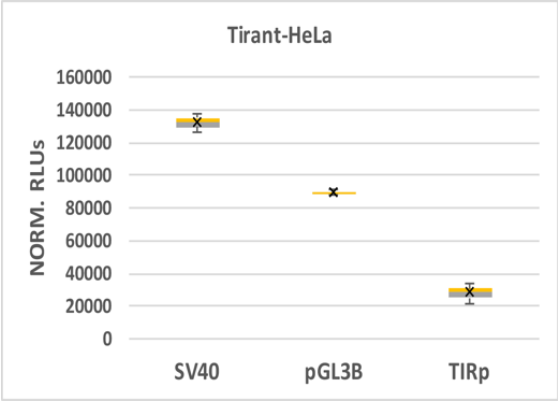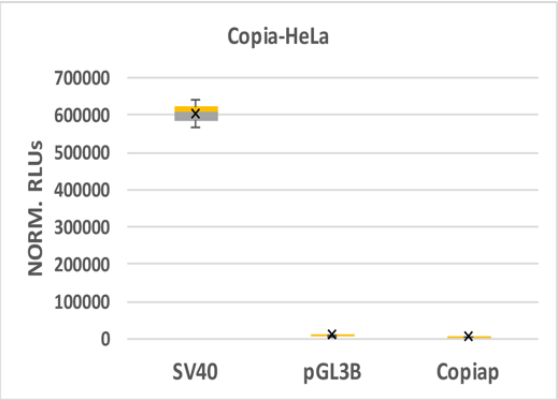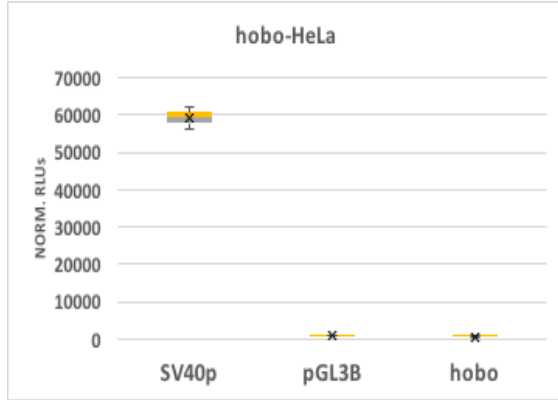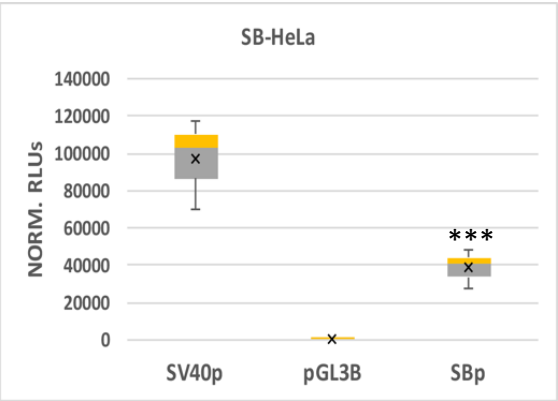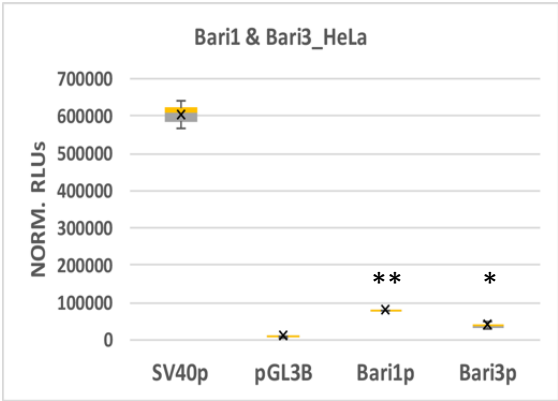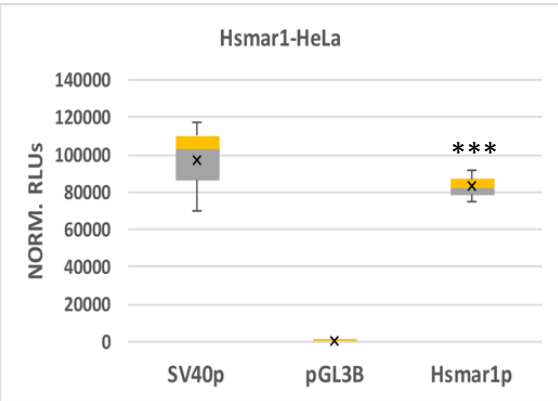

Supplement: Supplementary file 5 — Figure S1. Luciferase-promoter assay in HeLa cells. Individual promoter-luciferase assay results in HeLa cells. Top and bottom whiskers: maximum and minimum values of each samples respectively. Top and bottom of boxes: 75th and 25th percentile of the samples respectively. Line through the boxes: median of each sample. X markers: mean of each samples. The statistical significance of promoter activity against the promoter-less cassette is shown. *P < 0.05; **P < 0.005; ***P < 0.001 (PDF 168 kb) [file 13100_2019_155_MOESM5_ESM.pdf]

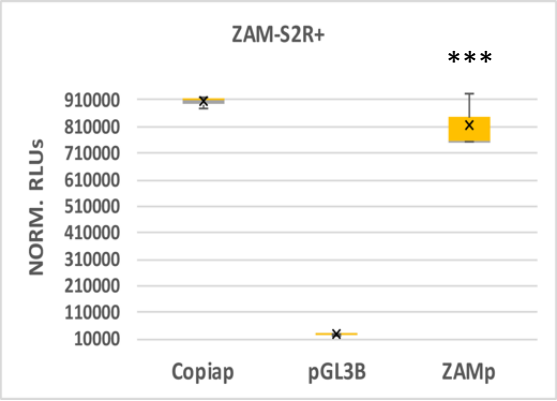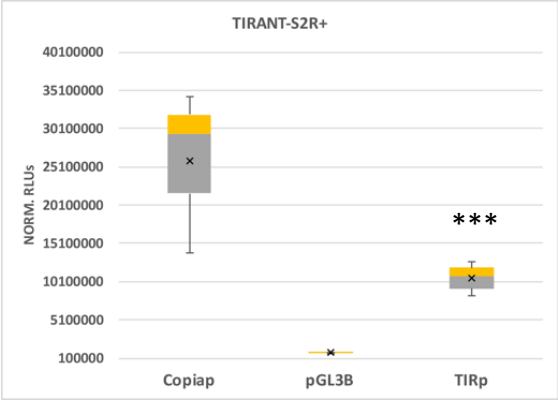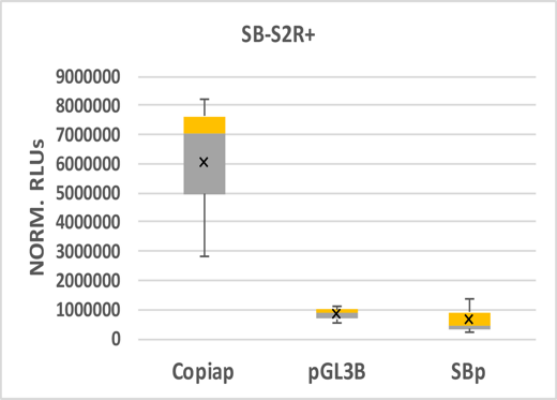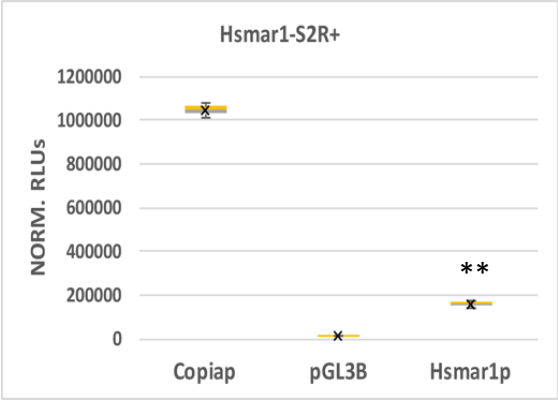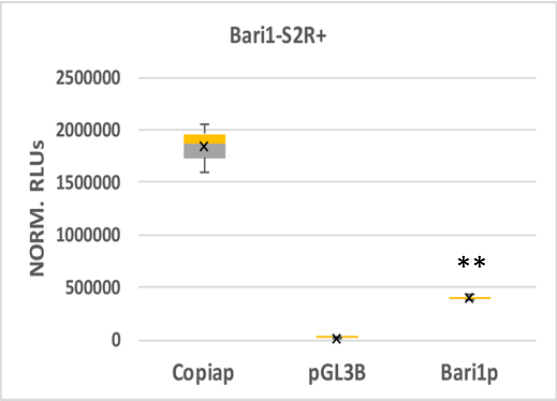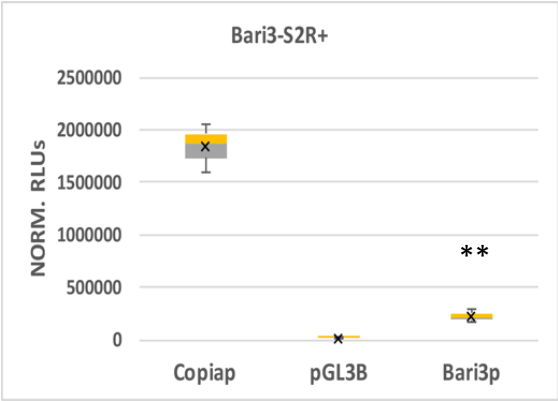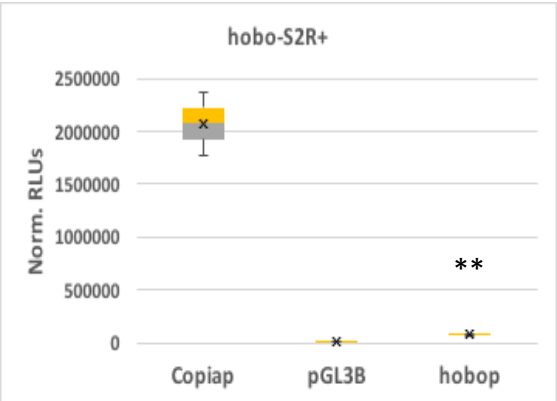

Supplement: Supplementary file 6 — Figure S2. Luciferase-promoter assay in S2R+ cells. Individual promoter-luciferase assay results in S2R+ cells of D. melanogaster. Top and bottom whiskers: maximum and minimum values of each samples respectively. Top and bottom of boxes: 75th and 25th percentile of the samples respectively. Line through the boxes: median of each sample. X markers: mean of each samples. The statistical significance of promoter activity against the promoter-less cassette is shown. **P < 0.005; ***P < 0.001 (PDF 174 kb) [file 13100_2019_155_MOESM6_ESM.pdf]

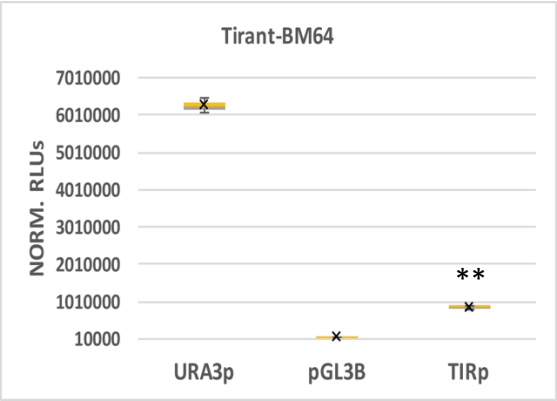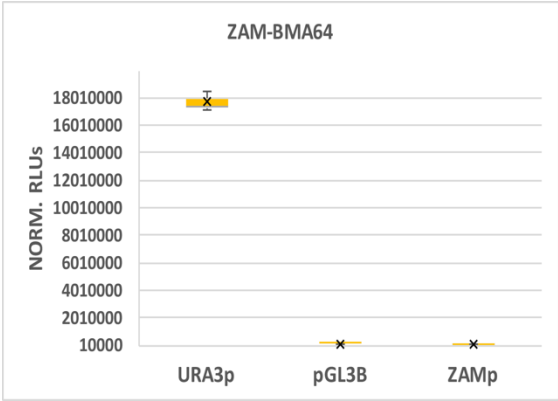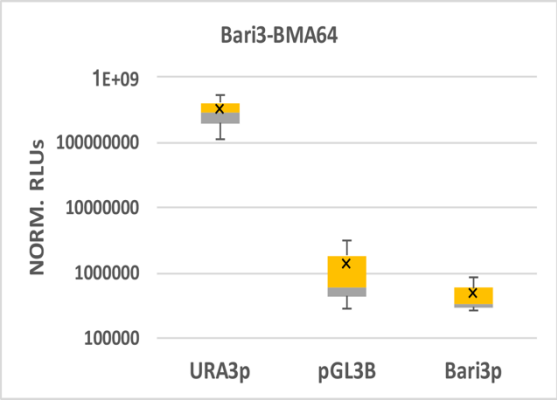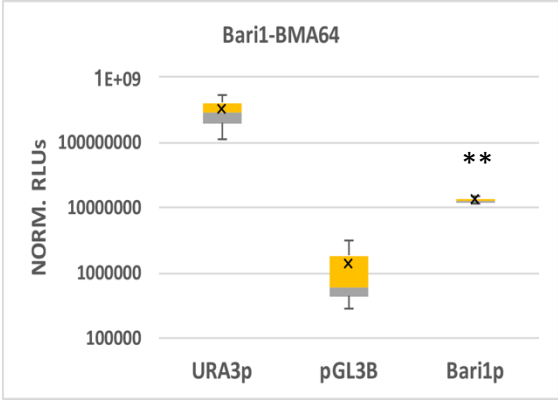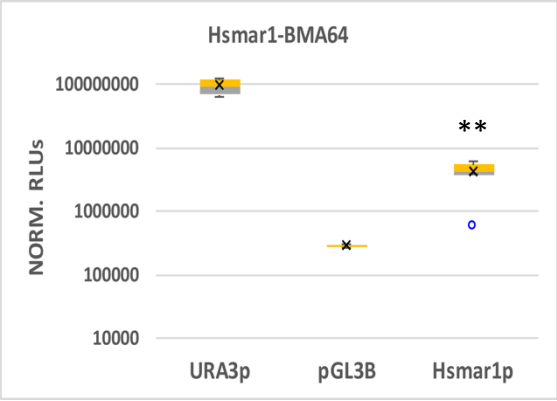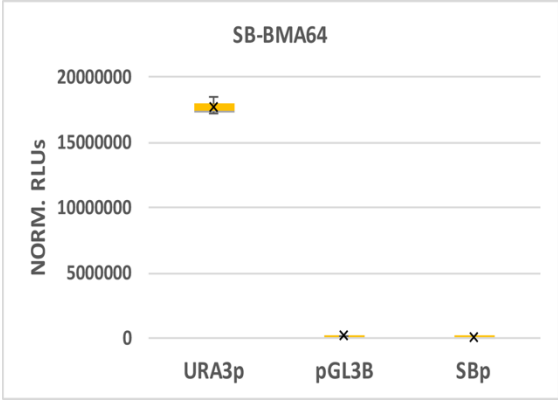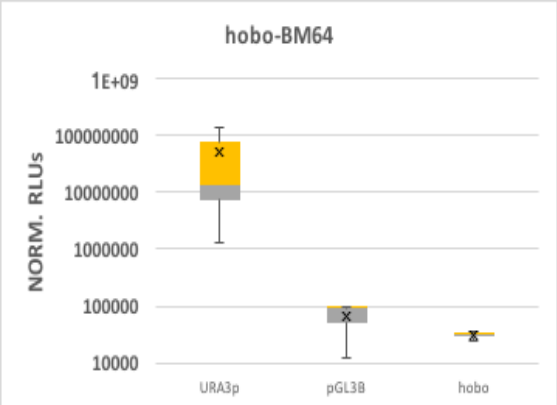

Supplement: Supplementary file 7 — Figure S3. Luciferase-promoter assay in S. cerevisiae cells (BMA64-1A). Individual promoter-luciferase assay results in yeast. Top and bottom whiskers: maximum and minimum values of each samples respectively. Top and bottom of boxes: 75th and 25th percentile of the samples respectively. Line through the boxes: median of each sample. X markers: mean of each samples. The statistical significance of promoter activity against the promoter-less cassette is shown. *P < 0.05; **P < 0.005 (PDF 216 kb) [file 13100_2019_155_MOESM7_ESM.pdf]

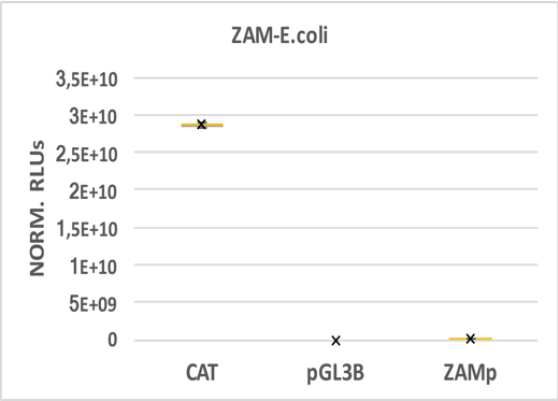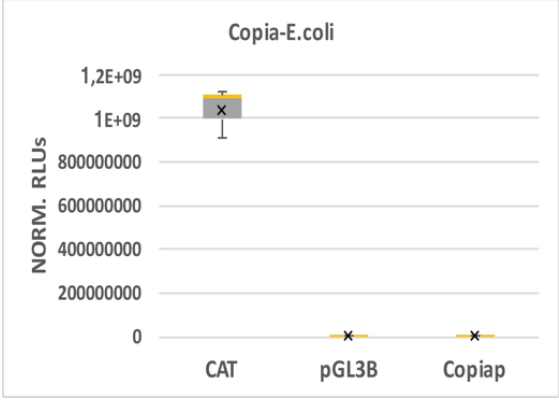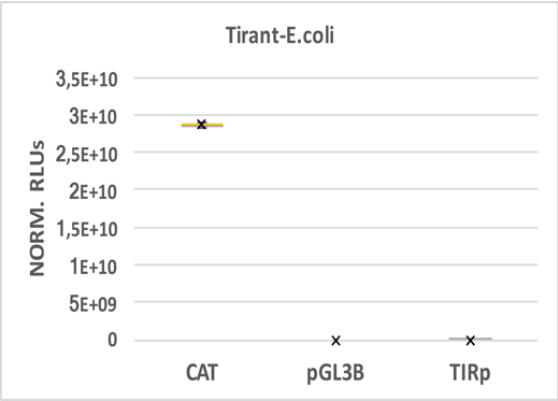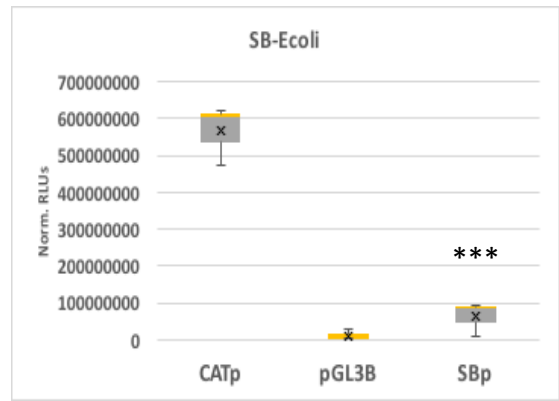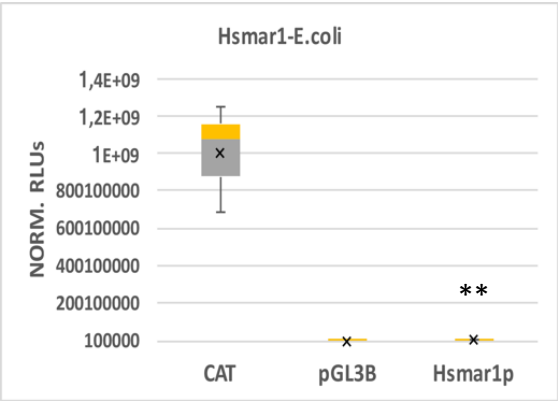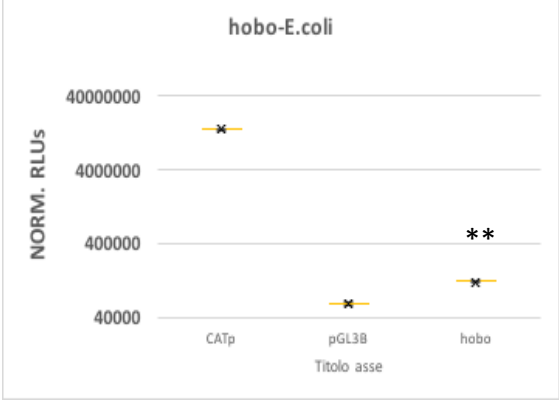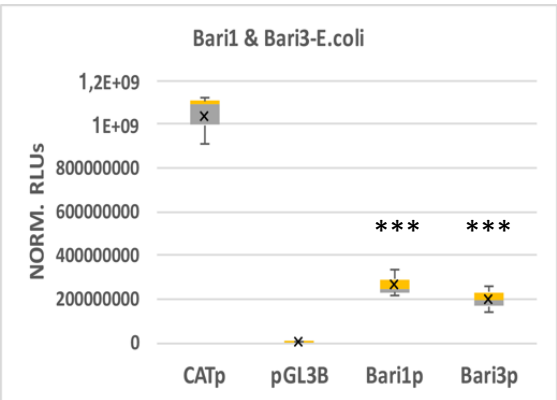

Supplement: Supplementary file 8 — Figure S4. Luciferase-promoter assay in bacteria (DH5alpha cells). Individual promoter-luciferase assay results in DH5alpha cells. Top and bottom whiskers: maximum and minimum values of each samples respectively. Top and bottom of boxes: 75th and 25th percentile of the samples respectively. Line through the boxes: median of each sample. X markers: mean of each samples. The statistical significance of promoter activity against the promoter-less cassette is shown. ***P < 0.001 (PDF 154 kb) [file 13100_2019_155_MOESM8_ESM.pdf]
